# Cue-based modulation of pain stimulus expectation: do ongoing oscillations reflect changes in pain perception? A registered report

**DOI:** 10.1098/rsos.240626

**Published:** 2024-06-19

**Authors:** Chiara Leu, Esther Glineur, Giulia Liberati

**Affiliations:** ^1^ Institute of Neuroscience, Université catholique de Louvain, Brussels, Belgium; ^2^ Université Lumière Lyon 2, Lyon, France; ^3^ Psychological Sciences Research Institute, Université catholique de Louvain, Louvain-la-Neuve, Belgium

**Keywords:** pain, nociception, neural oscillations, expectation, frequency-tagging

## Abstract

A promising stream of investigations is targeting ongoing neural oscillations and whether their modulation could be related to the perception of pain. Using an electroencephalography (EEG) frequency-tagging approach, sustained periodic thermonociceptive stimuli perceived as painful have been shown to modulate ongoing oscillations in the theta, alpha and beta bands at the frequency of stimulation. Nonetheless, it remains uncertain whether these modulations are indeed linked to pain perception. To test this relationship, we modulated pain perception using a cue-based expectation modulation paradigm and investigated whether ongoing oscillations in different frequency bands mirror the changes in stimulus perception. Forty healthy participants were instructed that a visual cue can precede either a high- or low-intensity stimulation. These cues were paired with three different levels of sustained periodic thermonociceptive stimuli (low, medium and high). Despite a strong effect of expectation on perceived stimulus intensity, this effect was not reflected in the modulation of the ongoing oscillations, suggesting a potential dissociation of pain perception and these oscillatory activities. Rather, it seems that the intensity of stimulation is the primary generator of the frequency-tagged EEG responses. Importantly, these results need to be confirmed by further investigations that could allow the detection of smaller effects than originally estimated.

## 1. Introduction

The synchronization of information across different brain regions through the flexible activity of ongoing neural oscillations has in recent years been associated with the processing of pain in the human brain [1]. Current investigations showcased the benefits of using an electroencephalography (EEG) frequency-tagging approach paired with the application of slow sustained periodic nociceptive stimuli for the exploration of the characteristics of pain-related ongoing oscillations [2–5]. In particular, this approach allows to differentiate between cortical activity related to the applied stimulus and unrelated activity by ‘tagging’ responses at the frequency of stimulation and its harmonics. As such, a periodic modulation was found at the frequency of stimulation in the aforementioned investigations in the alpha, beta and theta frequency bands. Expanding this approach to investigations using intracerebral EEG in patients undergoing a pre-surgical evaluation of focal epilepsy, Liberati *et al*. [6] found a preferential modulation of ongoing oscillations at the frequency of stimulation in the alpha and theta frequency bands following thermonociceptive stimulation in comparison with non-nociceptive vibrotactile stimuli. These results suggest that the modulation of ongoing oscillations could be related to nociception and/or the perception of pain. Yet, the functional relationship between ongoing oscillations and the perception of pain remains unclear. If there is in fact a link between these two factors, we expect that a modulation of pain perception should lead to a congruent change in the modulation of ongoing oscillations.

Expectation is a powerful cognitive modulation factor that can strongly influence the subjective experience of pain. While there are numerous ways to influence an individual’s expectation towards a painful stimulus, the modulation can be categorized into placebo analgesia, nocebo hyperalgesia and stimulus expectancies (reviewed by Atlas & Wager [7]). Importantly, while the former two categories rely on the application of an inert substance or intake of a fake drug, stimulus expectations achieve a modulation of pain perception solely by the association of pain-predictive cues [8–12].

To further understand the modulatory effects of expectation on pain perception, recent investigations studied its effects on ongoing neural oscillations. The application of a placebo analgesic as well as a nocebo hyperalgesic intervention both led to an increase in post-treatment resting-state alpha activity [13,14]. Even before the application of an expected painful stimulus, suppression of alpha frequency band activity has been observed in EEG as well as magnetoencephalography (MEG) investigations [15,16]. Similarly, a visual cue-based expectation modulation paradigm found a cluster of activity between 1 and 30 Hz when a painful stimulus was expected [17]. While similar results were found by Nickel *et al*. [18] regarding pre-stimulus activity in a predictive coding paradigm, changes in pain perception induced by expectation did not seem to have an effect on the modulation of ongoing oscillations. Another recent investigation found that, while expectations and prediction errors did not lead to any changes in local brain activity at the regions of interest (ROI), they did modulate the interregional connectivity within the chosen ROIs in the alpha and gamma frequency bands [19]. As discussed by Nickel *et al*. [18], it could be possible that commonly used approaches to analyse oscillatory activity are not sufficient to unravel the complexity of pain perception, as higher-order cortical processes such as the contextual modulation of pain might not be rigorously time-locked to the application of a painful stimulus. We aimed to overcome this limitation by using an EEG frequency-tagging approach, which allowed us to more clearly differentiate between activity related to the applied stimulus and other ongoing activity. Moreover, by using long-lasting periodic sustained stimuli, we aimed to capture high-level processes related to stimulus expectation to a larger extent than it is possible in the analysis of relatively brief and sudden stimuli.

We employed a cue-based stimulus expectation modulation paradigm to investigate whether changes in stimulus perception induced by expectation will lead to congruent changes in the modulation of ongoing oscillations at the frequency of stimulation and its harmonics. Based on Atlas *et al*. [8] and Keltner *et al.* [11], we expect (i) that the information (cue) presented to participants before a nociceptive stimulus can influence the expectations towards that stimulus and consequently (ii) alter the perception of this stimulus. Specifically, we hypothesized that if the same medium-intensity stimulus is presented with a cue indicating the following stimulus would have a low intensity, the rating of perception would be lower than if the same stimulus is presented with a cue indicating that the stimulus would be highly intense. As demonstrated in previous investigations from our lab [2,4–6], we expected (iii) the ultra-slow sustained thermonociceptive stimulation to elicit a periodic response in the different frequency bands at the frequency of stimulation and its harmonics. If the modulation of ongoing oscillations is indeed functionally related to pain perception, we hypothesized (iv) that the aggregated amplitudes at the frequency of interest (FOI) will exhibit a change in modulation congruent to the changes in stimulus perception induced by the cue-based expectation modulation. This would provide evidence that there is an association between the modulation of ongoing oscillations and pain perception.

## 2. Methods

All anonymized raw datasets and digital study materials are available in the public archive of Harvard Dataverse (10.7910/DVN/40ZRQR). Stage 1 recommendation and review history can be found here: https://rr.peercommunityin.org/articles/rec?id=432, while Stage 2 recommendation and review history is accesible here: https://rr.peercommunityin.org/articles/rec?id=675.

### 2.1. Participants

We recruited 40 healthy participants. A detailed sample size rationale as well as a discussion of the expected effect sizes can be found in the electronic supplementary material. Participants who have neurological diseases, psychiatric disorders or recent upper limb trauma upon direct questioning were excluded from the study. In addition, those who have taken paracetamol, non-steroidal anti-inflammatory drugs or acetylsalicylic acid within 12 h before the assessment were also excluded. Before the assessment began, written informed consent was obtained from all participants, who were also informed that they had the option to withdraw from the study at any time. We recruited participants between the ages of 18 and 35 (mean ± s.d.: 23.65 ± 3.45), with the aim of achieving a gender-balanced sample size (17 males/ 23 females). Participants were recruited via an established Facebook group, as well as posters on campus and word-of-mouth.

All procedures were performed in accordance with the relevant guidelines and regulations. The local Research Ethics Committee approved all experimental procedures (Commission d’Ethique Hospitalo-Facultaire Saint-Luc UCLouvain, B403201316436).

### 2.2. Sample size calculation

Previous investigations in this lab have shown that 15–20 participants are sufficient to observe the modulation of neural oscillations induced by a sustained periodic nociceptive stimulation [2,5]. This is largely owing to the high signal-to-noise ratio in the periodic responses to the ultra-slow 0.2 Hz sustained periodic stimulation, which can even be differentiated from noise at an individual level [2]. Other investigations using cue-based expectation modulation while acquiring EEG data recruited between 10 and 20 participants per experiment [8,9,11,13,20] and more recent investigations recruited between 40 and 48 participants [18,19].

We thus used a simulation-based approach to calculate appropriate power and sample size estimation to reach sufficient statistical power and detect a specific effect in a linear mixed model (LMM). The calculations were conducted using R Statistical Software (v. 4.1.0, [21]) and the R package ‘simr’ [22]. The regression model used for the simulated LMM was built as follows: amplitude ~ temperature + cue + temperature:cue + (one|subject), as detailed in our hypotheses plan and statistical analysis section. The simulated model is based on Mulders *et al*. [5]. This publication was chosen since the same stimulation and analysis techniques (i.e. frequency tagging of ongoing oscillations) as proposed in this investigation were used to analyse differences in the modulation of ongoing oscillations induced by different stimulation surface areas. The LMM interaction between temperature and surface in their investigation had an intermediate effect size of *η* ^2^_*p*_ = 0.060 for the phase-locked response. We simulated the LMM based on the mean and s.d. obtained for the phase-locked response using a small-variable surface of the contact-heat thermode probe for the stimulation (equalling our HH condition, mean = 0.59 µV, s.d. = 0.33 µV) and a small-fixed surface of stimulation (equalling our LL condition, mean = 0.41 µV, s.d. = 0.31 µV). The values for the medium-intensity conditions (HM (mean = 0.545 µV, s.d. = 0.349 µV) and LM (mean = 0.455 µV, s.d. = 0.291 µV)) were estimated based on the percentual difference in rating between these conditions that we observed in our behavioural pilot study (18%) (see electronic supplementary material). This percentage is similar to the difference observed in the ratings between HM and LM conditions in Atlas *et al*. [8]. We therefore calculated the mean between our chosen HH and LL values, lowered it by 9% for the condition LM and increased it by 9% for the condition HM. These values reflect our assumption that a stimulus that is expected to be more painful will lead to a larger amplitude at the frequency of stimulation and vice versa. The output of this LMM (based on intercept (0.809), slopes for temperature, cue and interaction (−0.228, −0.483 and 0.444), residual variance (0.107) and random intercept (0)) was then fed to the LMM-specific sample size simulation.

In the power estimation, we specifically tested for the interaction effect between temperature and cue, since this is our main comparison of interest. Additionally, interactions usually have a lower effect size compared with main effects and are therefore more critical for the calculation of the adequate sample size. According to the sample size simulation, a sample size of 25 participants would enable us to reach a statistical power of 0.9 while using an alpha level of 0.02. To avoid missing out on any effect and to account for the potential exclusion of participants from the final data analysis (e.g. owing to artefacts in the EEG signal), we decided to increase the sample size to 40 participants.

We considered recruiting a sample that would inform us not only about the effect size of interest but that would also be able to detect the smallest effect that one could possibly be interested in [23]. The necessary sample size was calculated by obtaining the 80% confidence interval (CI) of the LMM and replacing the model estimates with the lower bound estimates of the CI. This updated model was used for the simulation of the power-to-sample size relationship, resulting in a recommendation to test 150 participants. Unfortunately, limited resources do not allow us to test such a large cohort, and we decided to test only for the more conventional effect size of interest. In consequence, a non-significant result in the LMMs will not necessarily prove the absence of an effect but could also be owing to the sample size that might not be large enough to detect effects that are smaller than expected (as noted in the table 1).

**Table 1.** Hypotheses table defined prior to data collection, detailing hypotheses, analysis plans and interpretations given different outcomes.Further information on the sampling plan can be found in §2.2 and the electronic supplementary material.

question	hypothesis	sampling plan (power analysis)	analysis plan	interpretation given different outcomes	outcome
*behavioural response*					
does the intensity cue influence the rating of expected pain?	a high pain cue will lead to a higher expected pain rating than a low pain cue	see below. Expected detectable effect size is around *η* ^2^ = 0.058 (see electronic supplementary material for in-depth justifications).	rating_expected = cue + (one|subject) DV: expected *i*ntensity rating IV: cue random coefficient: subject	positive control: if the rating matches our expectations, we confirm that the cue is influencing pain expectations as intended. If not, the experiment cannot be used.	hypothesis confirmed
do different cues differentially influence the perception of the same painful stimulus?	a medium pain trial paired with a high-intensity cue will lead to a higher perceived intensity rating than a medium pain trial paired with a low-intensity cue	see below. Expected detectable effect size is around *η* ^2^ = 0.058 (see electronic supplementary material for in-depth justifications).	rating_perceived = temperature × cue + (one|subject) DV: perceived intensity rating IV: temperature, cue random coefficient: subject	positive control: a correct hypothesis would confirm that the expectation shapes the perception of the cue-associated stimuli. A dissociation of expectation and perception indicates that the cue-based paradigm was not successful at changing subjective intensity perception.	hypothesis confirmed
*time-locked, phase-locked response*					
does the sustained periodic stimulation lead to a periodic EEG modulation at the frequency of interest?	the slow sustained periodic stimulation will elicit a periodic response at the frequency of interest	see below. This sample size will allow us to detect an estimated effect size of around *η* ^2^ = 0.083 (see electronic supplementary material for in-depth justifications).	multi-sensor cluster-based permutation Wilcoxon signed-rank test (Bonferroni corrected) of aggregated and averaged amplitudes at FOIs	a periodic response shows that the stimulation paradigm induces the expected neural activity. Since the sample size is not sufficient to detect the smallest possible effect one would still be interested in, no definitive conclusions will be drawn from a non-significant result.	hypothesis confirmed only for condition HH; no definitive conclusion is possible (see sample size limitation)
does a cue-based expectation task modulate the EEG signal at the FOI in the frequency domain?	the amplitude at the FOI induced by the medium-intensity stimulation will exhibit a larger modulation following a cue indicating high intensity than a cue for low intensity	to reach a statistical power of 0.9 with an alpha level of 0.02, 40 participants will be enrolled. Calculations are based on power simulations using the simr package in R [22]. This sample size will allow us to detect an effect around *η* ^2^_*p*_ = 0.09 (see electronic supplementary material for in-depth justifications).	amplitude_FOI = temperature × cue + (one|subject) DV: amplitude at the FOI IV: temperature, cue random coefficient: subject	a change in modulation congruent to the change in intensity perception would reveal a possible connection between perceived pain and ongoing oscillations. Since the sample size is not sufficient to detect the smallest possible effect one would still be interested in, no definitive conclusions will be drawn from a non-significant result.	hypothesis disconfirmed; no definitive conclusion possible (see sample size limitation)
*time-locked, non-phase-locked response*					
does a slow sustained periodic stimulation lead to a periodic neural response at the frequency of interest for the different frequency bands?	a periodic modulation will be elicited in all frequency bands	see above. This sample size will allow us to detect an effect size of *η* ^2^ = 0.083 (see electronic supplementary material for in-depth justifications).	multi-sensor cluster-based permutation and Wilcoxon signed-rank test of aggregated and averaged amplitudes at FOIs in the different frequency bands^a^	a modulation at the frequency of stimulation indicates that sustained periodic stimulation leads to a periodic response also in the different frequency bands [2]. Since the sample size is not sufficient to detect the smallest possible effect one would still be interested in, no definitive conclusions will be drawn from a non-significant result.	hypothesis confirmed only for condition HH; no definitive conclusion possible (see sample size limitation).
does a cue-based expectation task modulate the ongoing oscillations in the different frequency bands in the time–frequency domain?	the medium-intensity stimulation will lead to a larger modulation of amplitude at the FOI following a high-intensity cue compared with the same stimulation followed by a cue for low intensity	see above. This sample size will allow us to detect an effect size around *η* ^2^_*p*_ = 0.09 (see electronic supplementary material for in-depth justifications).	amplitude_FOI_OO^a^ = temperature × cue + (one|subject) DV: amplitude at the FOI for each frequency band IV: temperature, cue random coefficient: subject	a modulation of ongoing oscillations mirroring the level of intensity suggested by the cue would suggest that pain rating and ongoing oscillations might be functionally connected. Since the sample size is not sufficient to detect the smallest possible effect one would still be interested in (*n* = 150) [23], a non-significant result of this statistical test does not necessarily indicate that there is a definitive absence of an effect and no definitive conclusions will be drawn from a non-significant result.	hypothesis disconfirmed; no definitive conclusion possible (see sample size limitation).

^a^One test for each frequency band (theta, alpha and beta)

amplitude_FOI, amplitude of phase-locked neural response; amplitude_FOI_OO, amplitude of non-phase-locked neural response for each frequency band (theta, alpha, beta); DV, dependent variable; FOI, frequency of interest; IV, independent variable; LMM, linear mixed mode.

### 2.3. Thermonociceptive stimulation

**Figure 1.**
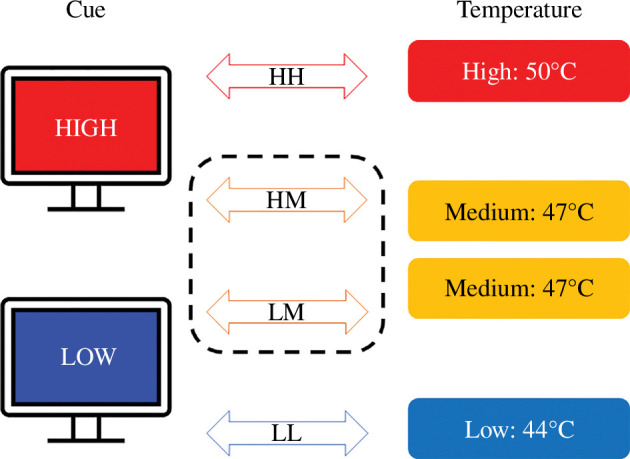
Cue-based expectation modulation paradigm, adapted from Atlas *et al*. [8]. Cues indicating a high-, respectively, low-intensity stimulus were adapted from Keltner *et al*. [11].

Thermonociceptive stimuli were delivered using a thermal cutaneous stimulator (TCS II, QST.Lab, Strasbourg, France) with the square T11 probe, which is set with five micro-Peltier elements (each approx. 181 mm^2^) whose temperature can vary at rates of up to 75°C s^−1^ and which can be activated individually. The full surface was used in this experiment, covering a rectangular area of 9 cm^2^. A sustained periodic stimulation with a frequency of 0.2 Hz was applied and the baseline temperature of stimulation was set to 35°C. The peaks of the stimulation varied from 44°C for the low-intensity condition, over 47°C for the medium-intensity condition to 50°C for the high-intensity condition (illustrated in figure 1). Each sustained periodic stimulation comprised 10 stimulation cycles, lasting a total of 10 × 5 s per stimulus, similar to Liberati *et al*. [6], Mulders *et al*. [5] and Leu *et al*. [4]. Shorter cycle durations were chosen compared with previous investigations to avoid subjecting the participants to a large number of thermonociceptive stimuli. Inter-stimulus intervals were variable and self-paced by the experimenter to allow participants to provide intensity ratings. The thermode was placed on the volar forearm of the dominant arm of the participants and was displaced after each trial to avoid habituation or sensitization.

### 2.4. Experimental procedure

#### 2.4.1. Expectation cue

**Figure 2.**
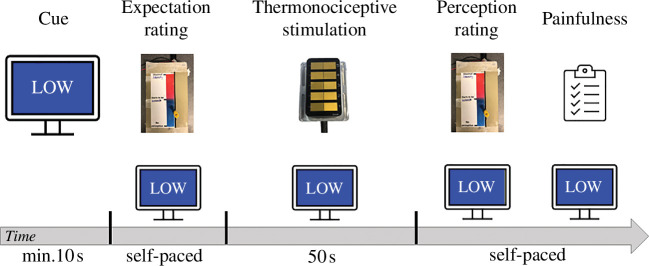
Trial design for one example stimulus, using a cue for a low intensity. Visual analogue scale (VAS) ratings were given on a scale from min: ‘no perception’ to a max: ‘most intense perception imaginable’. Participants were asked to evaluate the painfulness of the stimulus with a simple yes/no answer.

The visual cues, adapted from Keltner *et al*. [11], were displayed on a monitor in front of the participants. The cues consisted of a coloured square (blue for low-intensity stimulation and red for high-intensity stimulation), covering the full screen. In the middle of the square, the word ‘low’ or ‘high’, respectively, was displayed (illustrated in figure 1). The participants received verbal instructions identifying each cue and which stimulus intensity it is associated with. The cue was presented to the participants prior to the onset of each stimulus and remained visible during the stimulation (illustrated in figure 2). A pilot study was conducted prior to data collection to ensure that the chosen paradigm would be able to modulate subjects’ pain perception (see electronic supplementary material).

#### 2.4.2. Cue-based expectation modulation

Five blocks of stimuli were implemented, each block consisting of eight trials (each trial consisted of 50 s of sustained periodic stimulation), adapted from Atlas *et al*. [8]. The first block was used to establish the link between the expectation cue and the stimulation temperature and consisted of only four trials, which were not considered for the analysis. Therefore, in this block, the cue for low intensity was always paired with a low intensity stimulus (LL) and the cue for high intensity was always paired with a high-intensity stimulus (HH). The second block also started with two trials of matching conditions (LL/HH), followed by a randomized sequence of trials including unmatched cue/temperature combinations. In the unmatched conditions, medium-intensity stimuli were paired with either a cue for high intensity (HM) or a cue for low intensity (LM). Blocks three to five were random in the sequence of conditions. In each block, each condition needed to be presented two times, resulting in a total of eight trials per condition for the analysis. The experimenters (as well as the participants) were blinded regarding the condition that was to be applied; thus, neither knew whether the current stimulus was a matched or unmatched condition.

### 2.5. Behavioural measures

Participants rated the expected intensity of stimulation on a visual analogue scale (VAS) using a 10 cm ungraduated sliding ruler right after seeing the cue before the beginning of the stimulation. The lower extremity of the VAS was labelled ‘no perception’, and the higher extremity was labelled ‘the most intense perception you can imagine’. The time interval between the rating and the start of the stimulation was variable. During the thermonociceptive stimulation, participants were instructed to sit as still as possible to generate an artefact-free EEG signal. After the stimulation, participants heard a beep sound, which indicated the end of the trial. Participants then had to indicate on the VAS how intense they perceived the thermonociceptive stimulation overall, as well as whether they perceived the stimulation as painful or not (as illustrated in figure 2).

### 2.6. Electroencephalography recordings

EEG was recorded using an elastic electrode cap with 64 active, pre-amplified Ag–AgCl electrodes (BioSemi, The Netherlands), which were arranged according to the international 10–10 system. To ensure a clean signal, the direct-current offset was kept below 30 mV. All electrodes were re-referenced offline to the average electrode activity. The recorded signal was stored in the BioSemi ActiView software for offline analyses.

### 2.7. Electroencephalography analysis

The EEG recordings were analysed using the Letswave7 (https://www.letswave.org/) toolbox in MATLAB (2022a, The MathWorks).

#### 2.7.1. Analysis of the phase-locked response

To isolate activity related to the applied stimulus, we made use of the frequency-tagging analysis approach [24]. According to the rationale of this approach, a periodic stimulation elicits a periodic activation of higher-order neurons, which in turn leads to a periodic EEG response at the frequency of stimulation and its harmonics [3,25]. This approach has been used extensively in our lab over the past years [2–6,26], leading to a standardized analysis approach: first, events were created based on the triggers related to the onset of the stimulation. Each trigger received a label according to the condition it preceded (HH, LL, HM and LM). Then, slow drift and high-frequency power line noise were removed using a Butterworth band-pass filter between 0.05 and 40 Hz. Epochs were segmented into segments of 0–50 s, relative to the onset of the stimulation, creating one file per event code containing all eight trials of this condition. Electrodes P9, P10 and Iz were removed, since owing to their placement on the EEG cap, they frequently only record muscular noise rather than brain activity. All signals were re-referenced to the average of the remaining electrode set. Then, an independent component analysis (Fast ICA algorithm) [27] was employed to detect artefacts owing to eye movement or other muscular artefacts and remove them. The ICA was computed for each subject separately across all conditions at the same time, using the ‘runica’ algorithm [28], decomposing the full rank data matrix into 61 independent components. Therefore, the same components were removed in each condition for each subject. Additionally, trials with an amplitude larger than ±500 μV [26] on any of the electrodes were excluded. Any participant with less than five trials at this stage would have been removed from the dataset, but this did not occur. Finally, the average signal was calculated for each participant and each condition and then transformed into the frequency domain using a discrete fast Fourier transform [22]. Residual noise was partially removed by subtracting the average amplitude of the signal measures at two–five neighbouring frequencies, at each electrode and at each frequency bin.

Since the periodic response elicited by the ultra-slow sustained periodic stimulation is not a perfect sinewave, the peaks in the amplitude of the frequency spectrum do not only appear at the frequency of stimulation itself but also at its harmonics. To account for this, the amplitude at the frequency of stimulation and its harmonics was aggregated, and the resulting amplitude at the FOI was used for the statistical analysis. To aggregate the harmonics, the signal was cut into chunks of 0.2 Hz length, starting at 0.1 Hz. Therefore, in each chunk, the signal in the middle corresponds to the harmonic of the frequency of stimulation. Then, the chunks were averaged, and the resulting amplitude was multiplied by the number of chunks for which the average has been calculated. The whole electrode set was taken into account for this procedure.

#### 2.7.2. Analysis of the modulation of ongoing oscillations

The analysis of the modulation of ongoing oscillations was almost identical to the previously outlined analysis. To investigate the effect of our stimulation on the periodic modulation of the amplitude of ongoing neural oscillations within different frequency bands (theta: 4–8 Hz, alpha: 8–12 Hz and beta: 12–40 Hz), the EEG signal was additionally filtered using a fourth-order Butterworth filter for each frequency band after calculating the ICA and re-referencing of the electrodes in the remaining signal. Another additional step was the estimation of the envelopes of the signal, which was computed using a Hilbert transform. The following steps were equal to the procedure described for the phase-locked response, including the aggregation of the signal amplitudes at the frequency of stimulation and its harmonics. The amplitude at the FOI was used for the statistical analysis in each frequency band and the whole electrode set was considered.

### 2.8. Statistical analysis

Statistical analysis was done using R Statistical Software (v. 4.1.0, [21]) and MATLAB (2020b, The MathWorks). The significance level of *p* < 0.05 was set for the behavioural analysis and LMMs. The LMM was fitted using REML, and a Kenward–Roger approximation to produce appropriate type I error rates for smaller sample sizes was used to test the significance of the results. All explicit formulae/equations for the statistical analysis can be found in table 1.

#### 2.8.1. Behavioural data

To assess whether the cue affected the rating of expected stimulus intensity, an LMM with the independent variable (IV) cue and dependent variable (DV) expectation rating was used. The factor subject accounted for the variation of the regression model intercept across participants and was therefore a random effect in the model. This model was the positive control for the factor cue; if the cue would not be effective in influencing the expected stimulus intensity, we had to assume that the cue-based expectation modulation paradigm in this experiment failed.

Furthermore, we employed another LMM to analyse the effect of cue (two levels: low and high) and stimulation temperature (two levels: matched and unmatched) (IVs) on the rating of perceived stimulus intensity perception (DV). We further wanted to assess the interaction between these two factors on the intensity rating. As in the aforementioned LMM, the subject was used as a random effect. We hypothesized that the medium-intensity stimulation paired with the high-intensity cue (HM) would lead to a higher rating of perceived stimulus intensity compared with the medium stimulus paired with the low-intensity cue (LM).

#### 2.8.2. Periodic response

To assess whether the amplitude at the FOI was significantly different from zero, a right-tailed multi-sensor cluster-based permutation test using the Wilcoxon signed-rank test as test statistic was used. To do this, for each condition, the corresponding data were merged into one file, containing all participants. The test compared each signal with 0, using a Bonferroni corrected alpha level of 0.0125 (the standard alpha level 0.05 divided by the number of conditions). The threshold for the cluster-based permutation was also set to 0.0125, and 2000 permutations were computed. The multi-sensor analysis was set to a threshold of 0.161, which sets the threshold for the sensor connection so each channel has four neighbours on average. This approach allowed to control for a non-normal distribution of the data while taking potential type I error inflation owing to multiple testing into account. The electrode with the highest test statistic will be chosen for further analysis.

Based on previous results from this laboratory [2,4,5], we expected a periodic response in the EEG signal elicited by the sustained periodic stimulation significantly larger than zero.

To investigate whether the high-intensity cue paired with the medium-intensity stimulation (HM) would lead to a higher amplitude at the FOI for the phase-locked EEG signal compared with the low-intensity cue paired with the medium-intensity stimulation (LM), we used an LMM with the following factors: stimulation temperature and cue as independent (fixed) variables (IV) with an interaction, while the subject was a random factor. The amplitude at the FOI was used as DV.

#### 2.8.3. Modulation of ongoing oscillations

As for the phase-locked signal, we examined whether the amplitude at the FOI was significantly larger than zero with a right-tailed multi-sensor cluster-based permutation test using the Wilcoxon signed-rank test as a test statistic. The electrode with the highest test statistic was used for the continuation of the analysis.

We expected the amplitude at the FOI to be significantly larger than zero in all frequency bands [2,4,6]. To test our main hypotheses, we used LMM with the same structure as described above. The IVs were temperature and cue, while the subject was added as a random factor, accounting for the variation in the regression model between participants. Finally, the amplitude was the DV in this model. A separate LMM was calculated for the amplitude at the FOI in each frequency band.

We hypothesized that the amplitude at the FOI would be larger if the medium-intensity stimulation was preceded by a high-intensity cue (HM) compared with a low-intensity cue (LM). If this was the case, and the cue-based expectation modulation would change intensity perception in the same direction, these results would suggest that the modulation of ongoing oscillation could be functionally related to pain perception.

#### 2.8.4. Outliers

Any participant unable to complete data acquisition would have been excluded from the analysis. This was not the case in our dataset. Furthermore, any data points that violated the LMM assumptions after fitting the LMM were identified using a Shapiro–Wilk test to assess the normal distribution of the data as well as a Levene’s test, testing the dataset for homoscedasticity. If the data did not conform to normality, a log transform was applied, which conforms data to the assumption of normality by correcting right-skewed data into a more normal form [29]. Any data point that still violated any of the assumptions after the transformation or disproportionately affected the dataset after fitting the LMM was removed from the dataset and was not replaced. To ensure that we will still reach the targeted sample size and statistical power, a slightly larger group of participants will be recruited than required by the sample size calculation. Additionally, data points that over-proportionally influence the dataset were identified using Cook’s distance (*D*). This method calculates how much the fitted values of a given dataset change if just one data point is removed. The influence of a data point is expressed in the ‘distance’ *D*; the larger it is, the more influential the data point [30]. Therefore, any data point exceeding a *D* of 1 was removed from the dataset. Cook’s distance was calculated for each data point within a condition. Thus, for each condition and frequency band, a separate calculation will be done. Overall, data points exceeding the threshold for Cook’s distance were identified only in the phase-locked response. The results without removed outliers are reported in the electronic supplementary material.

## 3. Results

### 3.1. Behavioural results

**Figure 3.**
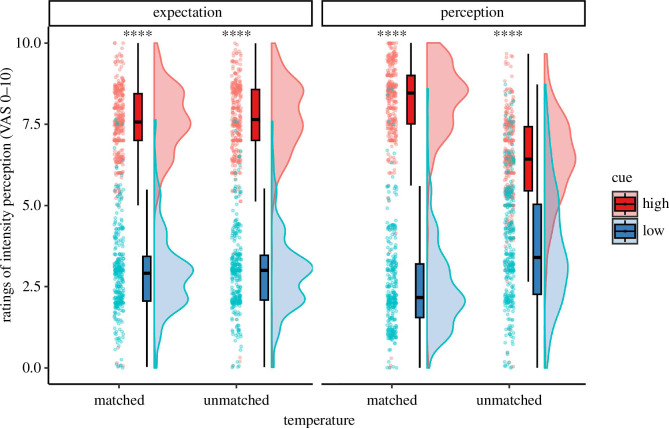
Ratings of expectation (left) and perception (right) collected on a VAS before and after each stimulation, illustrated in Raincloud plots [31]. Dots indicate single-subject ratings. Results of the *post hoc* pairwise comparisons are indicated at the top; ****= p < 0.001. ‘Matched’ refers to conditions HH and LL, while ‘unmatched’ refers to conditions HM and LM (cue/temperature).

**Figure 4.**
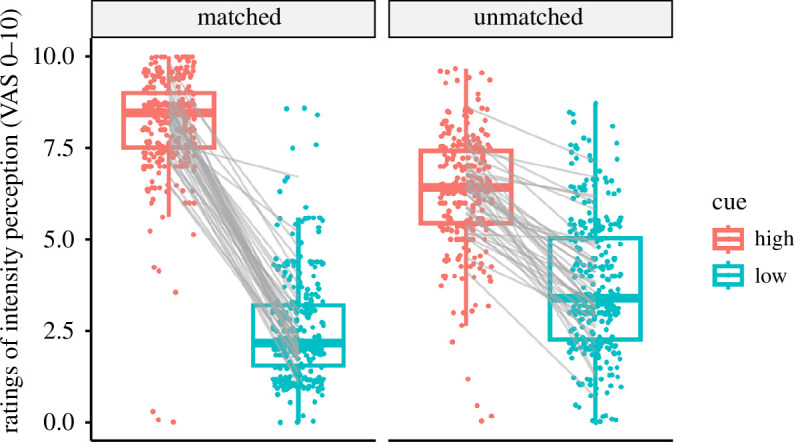
Ratings of intensity perception for each subject in each condition, given on a VAS. Grey lines connect the mean ratings of the same subject. Matched conditions equal the conditions HH and LL, while unmatched conditions represent HM and LM (cue/temperature).

The cue significantly influenced the expectation of the participants regarding the upcoming thermonociceptive stimulation (*F* (1, 1239) = 6108, *p* < 0.001, *η* ^2^_*p*_ = 0.83). The perception of the stimulation was also significantly modulated by both cue and stimulation temperature, as well as their interaction (table 2 and figure 3). *Post hoc* pairwise comparisons revealed that the medium-intensity stimulation was perceived as significantly more intense when preceded by a high-intensity cue compared with a low-intensity cue (*t* (1237) = 25.950, *p* < 0.001). Figure 4 illustrates the perception ratings for each participant in all conditions. Overall, stimuli were perceived as painful in condition HH (78.75% of trials), while this was rarely the case for condition LL (1.25% of trials). The medium-intensity condition was perceived as mostly non-painful when preceded by a low-intensity cue (LM, 9.7% rated as painful). Yet, the same stimulation preceded by a high-intensity cue (HM) was rated as painful in more than twice as many trials (22.8% of trials).

**Table 2.** Results of the linear mixed model with the formula DV *~ c*ue + condition + cue: condition + (one|subject).

**dependent variable**	cue			condition			interaction			
	*F*-value	*p*	*η^2^_p_* (95% CI)	*F*-value	*p*	*η^2^_p_* (95% CI)		*F*-value	*p*	*η^2^_p_* (95% CI)
perception ratings	(1,1237) 3357.926	**<0.001**	0.73 (0.71, 1)	20.634	**<0.001**	0.02 (0.01, 1)		451.532	<0.001*	0.27 (0.23, 1)
phase-locked response	(1,114.68) 7.912	**0.006**	0.06 (0.01, 1)	6.36	**0.013**	0.05 (0.01, 1)		26.026	**<0.001**	0.18 (0.09, 1)
theta	(1,117) 4.992	**0.027**	0.04 (0.00, 1)	4.588	**0.034**	0.04 (0.00, 1)		2.248	0.147	0.02 (0.00, 1)
alpha	(1,117) 10.761	**0.001**	0.08 (0.02, 1)	4.470	**0.037**	0.04 (0.00 ,1)		8.861	**0.004**	0.07 (0.01, 1)
beta	(1,117) 10.374	**0.002**	0.08 (0.02, 1)	1.921	0.168	0.02 (0.00, 1)		13.784	**<0.001**	0.11 (0.03, 1)

Significant *p* values are highlighted in bold font.

*η* ^2^_*p*_ was calculated as a measure of the partial effect size of each fixed effect, including its 95% CI.

Asterisks mark interactions that showed a significant difference in the *post hoc* pairwise comparison between the conditions HM and LM (which used the same stimulus intensity, the only difference being the concomitantly presented cue).

Electrodes of interest: phase-locked: Fz, theta: C2, alpha: PO3, beta: Fcz

### 3.2. Electroencephalography

#### 3.2.1. Phase-locked response

The Wilcoxon signed-rank test showed that a periodic modulation on the phase-locked response was only found in the HH condition. No significant periodic modulation was found in any of the other conditions. The electrode with the largest modulation was Fz, which was subsequently chosen as the electrode of interest for the remaining analysis of the phase-locked response. The data points of one subject were partially removed because they were identified as outliers, even after using a log transform to increase the normality of the data distribution. Hence, statistical analysis was done on the log-transformed data. The original amplitudes are shown in all figures to increase the intuitive understanding of the graphs. The LMM showed a significant effect of the cue as well as of the temperature × cue interaction on the amplitude at the FOI (table 2). *Post hoc* pairwise comparison revealed a significant difference between the matched conditions (HH versus LL, *p* < 0.001), but no significant difference between the unmatched conditions (HM versus LM, *p* = 0.11). Further analysis revealed a significant difference between conditions HH and HM (*p* < 0.0001) but not between LM and LL (*p* = 0.073).

#### 3.2.2. Modulation of ongoing oscillations

**Figure 5.**
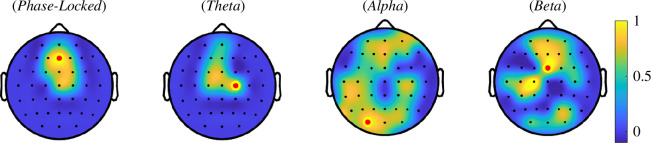
Topographies displaying the results of the multi-sensor cluster-based Wilcoxon signed-rank test for the condition HH (cue for high intensity + high-intensity stimulation). The electrode with the largest test statistic, which was used for the subsequent statistical analysis, is marked with a red dot.

Similar to the phase-locked response, only condition HH leads to a significant modulation at the FOI in the different frequency bands. The electrodes with the largest modulation in the theta, alpha and beta frequency bands were C2, PO3 and FCz. Scalp topographies illustrating the obtained Wilcoxon test statistics can be found in figure 5.

**Figure 6.**
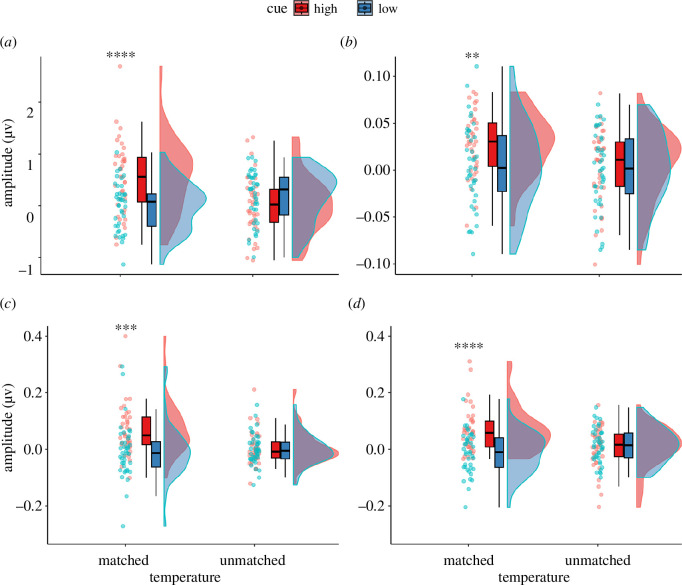
Raincloud plots [31] illustrating the amplitudes at the FOI for (*a*) *phase-locked response*, electrode Fz; (*b*) *theta frequency band* (4–8 Hz), electrode C2; (*c*) *alpha frequency band* (8–12 Hz), electrode PO3; and (*d*) *beta frequency band* (12–40 Hz), electrode FCz. ‘Matched’ refers to conditions HH and LL, while ‘unmatched’ refers to conditions HM and LM (cue/temperature). Significant differences in the *post hoc* paired *t*‐test are noted as follows: **= p < 0.01, ***= p < 0.001 and ****= p < 0.001.

As for the phase-locked response, all datasets were log-transformed to conform to the assumption of normality. While the statistical analysis was done on the log-transformed data, we chose to show the original amplitudes in the plots for enhanced readability. The LMMs in the different frequency bands all yielded similar results (table 2). While significant effects of cue, stimulus temperature and their interaction were observed in almost all frequency bands, all *post hoc* pairwise comparisons revealed that the difference in amplitude at the FOI was not significant between conditions that were presented using the same stimulation temperature (HM and LM). Yet, in all comparisons, condition HH led to significantly larger amplitudes at the FOI than condition LL (illustrated in figure 6). Additional pairwise comparisons revealed that in all frequency bands, condition HH elicited significantly larger amplitude at the FOI than condition HM (*p* = 0.011, *p* < 0.001 and *p* < 0.001 for theta, alpha and beta frequency bands, respectively), while no significant differences were observed between conditions LM and LL. Illustrations of the spectra up to 1 Hz for each frequency band can be found in the electronic supplementary material.

## 4. Discussion

The cue-based expectation modulation paradigm, which was adapted from Atlas *et al*. [8] and Keltner *et al*. [11], influenced the participants’ stimulus intensity expectation and perception as intended; both positive controls regarding the behavioural aspect of the experiment have been met. The visual cue changed the expectation of the participants towards the upcoming stimulus, and participants anticipated the correct level of stimulus intensity based on the cue they were given. Moreover, the cue also significantly changed the stimulus intensity perception of the participants in the medium-intensity stimulation trials: participants experienced the stimulation as more painful and intense when shown a cue indicating a high stimulus intensity compared with being shown a cue for LL. Consequently, given our hypothesis, we expected the modulation of ongoing oscillations to reflect these cue-based changes in stimulus perception during the stimuli delivered at a medium intensity (HM and LM).

A significant modulation of ongoing oscillations at the FOI was only found in the high-intensity stimulation condition. Additionally, while significant differences were found for the main effects and interactions between cue and stimulation temperature, *post hoc* pairwise comparisons uncovered that these differences were mainly driven by the conditions HH and LL; the conditions of interest HM and LM did not differ significantly in their modulation at the FOI for the specified electrodes in any of the analysed frequency bands. Moreover, significant differences in amplitude found between HH and HM indicate the intensity at which the stimulus is delivered is mainly contributing to the amplitude observed at the FOI, and not the expected level of stimulus intensity. This suggests a potential dissociation of stimulus intensity perception and the modulation of ongoing oscillations measured using scalp EEG.

While the frequency-tagging approach has been used numerous times in our laboratories, the present stimulation parameters diverged from previous experiments in their duration as well as in the stimulus temperatures. Notable, stimuli were shorter (10 instead of 15 cycles) and intensities lower (44°C and 47°C instead of only 50°C) compared with the previous publications using this technique [2,4,5]. While the numbers of cycles were carefully chosen following the re-analysis of the datasets of these previous investigations, it was less clear how much the stimulation temperature would affect the modulation of ongoing oscillations. As shown by Colon *et al*. [2], these slow sustained periodic stimuli recruit predominantly C-fibres, for which the activation threshold lies around 37–49°C [32,33]. Since we were not able to detect a significant modulation at the FOI for the low and medium stimulation intensities, one possible explanation would be that the temperatures were too low to recruit enough afferent fibres to elicit a periodic activation of higher-order neurons. Especially given the temperature of the LL, which was set just around the threshold for pain perception for most people, it seems reasonable that no large effects would be seen. Importantly, while we measured the neural responses to all four conditions, we were mainly interested in the responses to stimuli using the medium stimulation intensity (47°C). For conditions HM and LM, it was indeed rather surprising to not find a periodic response at the FOI. This might be a result of the lower stimulation temperature in combination with the very slow stimulation paradigm, as temporal summation (nociceptor discharge frequency greater than 0.5 Hz) is thought to be one of the means through which innocuous warm stimuli can activate nociceptive C-fibres [34]. While the low frequency of stimulation was chosen owing to its superior signal-to-noise ratio [2], a higher temperature for the medium-intensity trials might have led to a larger response at the FOI. Yet, as we tried to create a paradigm that had three clearly discernable stimulation temperatures that were also tolerable for the high-intensity stimulation, the range of temperatures to choose from for the paradigm was limited.

Nonetheless, while the response at the FOI might have not been significant for certain conditions, we could theoretically still observe the difference in the modulation of the ongoing oscillations at that frequency. Importantly, those differences will have to be interpreted with caution, as the observed responses were clearly rather small and not statistically different from zero. As expected, a significant difference between high- and low-intensity stimulation was found in all frequency bands, with the intensity of stimulation as the main differentiator. For both conditions, the expectations matched the applied stimulus. The comparison between the conditions preceded by the high expectation cue showed a similar pattern; the more intense stimulation led to a larger response at the frequency of stimulation in all frequency bands. Thus, neither the expectation of a high-intensity stimulus nor the mismatch between expected and perceived stimulus intensity for condition HM seemed to have an influence on the recorded amplitude.

On the contrary, the medium-intensity conditions (HM and LM) did not show any differences in their modulation, despite the strong effects the visual cues had on the intensity perception associated with the stimulation. While various investigations have found effects of pain stimulus expectation on EEG correlates [9,14,35–38], other recent publications found a similar dissociation between stimulus perception and oscillatory activity. Albu and Meagher [13] recorded an increase in oscillatory activity between 8 and 10 Hz related to thermonociceptive stimuli following a nocebo treatment. Yet, these changes were correlated to the catastrophizing of the individual and not the perceived pain intensity or unpleasantness. In a paradigm comparable to the one used in the present study, Nickel *et al*. [18] found a similar modulation of thermonociceptive stimulus perception based on visual cues but failed to detect related changes in either event-related potentials or oscillatory activity in their time–frequency analysis.

With our frequency-tagging approach, we aimed to overcome some of the pitfalls of other—more standard—EEG analyses, such as the focus on phase-locked responses and brief stimuli. However, it appears that scalp EEG might not have the specificity or spatial resolution to relay the complex network of activity that shapes human pain perception when the activity of a specific electrode is considered. Nevertheless, this does not imply that EEG cannot be used to assess relevant information on how neural activity guides pain perception.

On one hand, expanding traditional scalp EEG assessments to connectivity analyses and source modelling has been shown to be a promising tool to further understand the complex processes that integrate sensory and contextual information. Bott *et al*. [19] have provided interesting results using the same cue-based expectation paradigm as in a study by Nickel *et al*. [18], expanding the analysis from local oscillatory activity to interregional connectivity in a network of six pre-selected ROI associated with pain perception (such as contralateral primary somatosensory cortex, anterior cingulate and prefrontal cortices). While sensory information was mostly encoded in local activity, expectations were shaped exclusively by interregional connectivity, predominantly in the alpha frequency band from the prefrontal to the somatosensory cortex. These results highlight that the perception of pain, which depends on both sensory as well as contextual factors, can hardly be measured by a single electrode measured using scalp EEG. Thus, future EEG investigations should focus rather on the connectivity between different brain regions to further unravel how pain perception emerged from neural activity.

On the other hand, intracerebral EEG could overcome some limitations of scalp EEG. Previous investigations from our laboratory have shown that thermonociceptive stimuli (delivered in the same slow sustained periodic manner) are preferentially modulated in the human insula compared with non-nociceptive vibrotactile stimuli [6]. By using electrodes that are implanted in patients undergoing pre-surgical evaluation for focal refractory epilepsy, it is possible to assess the activity of brain regions that are difficult to assess using scalp EEG owing to the depth of their locations with a very high spatial as well as temporal resolution [39].

Finally, the statistical power given our sample size should be considered for the interpretation of our results. As outlined in table 2, while we had enough power (given the limitations of an alpha level of 0.02 and target power of 0.9) to find expected effect sizes based on previous investigations and our initial estimation, our sample size did not allow us to reach a statistical power that would allow us to find the ‘*smallest* possible effect that we would still be interested in’ [23]. Frequently, the targeted effect size is the observed effect in previous literature; yet, this approach might lead to the lack of support for hypotheses only because the effect might have been smaller than in previous investigations and not because there was truly no effect [23]. Additionally, since the amplitudes related to the medium-intensity stimulation were smaller than expected, this could have led us to overestimate the initial effect size. The large range of the CIs of the *post hoc* estimated effect sizes suggests that our effect size is not very precise, and a different investigation might yield different results. Thus, even though we did not find any modulations in the ongoing oscillations related to the differences in pain perception induced by the cue-based expectation modulation, we cannot fully reject this hypothesis based on our study alone and further investigations are needed to confirm our results.

## 5. Conclusion

Despite a strong effect of the visual cues on stimulus perception, no significant differences were observed in the modulation of ongoing oscillations at the FOI between the conditions of interest (medium-intensity stimulation preceded by either a cue for a high or a low stimulation intensity). These results could suggest a dissociation between stimulus perception and the modulation of ongoing oscillations measured using scalp EEG. While our results parallel other recent investigations using a cue-based expectation modulation, we cannot exclude that our sample size was not large enough to detect a potentially small effect, and definitive conclusions should not be drawn.

It appears that more advanced analysis procedures (such as source localization and connectivity analysis) or more spatially precise recordings of neural activity (such as iEEG) could be more beneficial to understand how our perception of pain emerges from neural activity.

## Data Availability

All anonymized raw datasets and digital study materials are available in the public archive of Harvard Dataverse (10.7910/DVN/40ZRQR). Electronic supplementary material is available online at [40].

## References

[1] Ploner M , Sorg C , Gross J . 2017 Brain rhythms of pain. Trends Cogn. Sci. **21** , 100–110. (10.1016/j.tics.2016.12.001) 28025007 PMC5374269

[2] Colon E , Liberati G , Mouraux A . 2017 EEG frequency tagging using ultra-slow periodic heat stimulation of the skin reveals cortical activity specifically related to C fiber thermonociceptors. Neuroimage **146** , 266–274, (10.1016/j.neuroimage.2016.11.045) 27871921 PMC5322834

[3] Colon E , Nozaradan S , Legrain V , Mouraux A . 2012 Steady-state evoked potentials to tag specific components of nociceptive cortical processing. Neuroimage **60** , 571–581. (10.1016/j.neuroimage.2011.12.015) 22197788

[4] Leu C , Courtin A , Cussac C , Liberati G . 2023 The role of ongoing oscillation in pain perception: absence of modulation by a concomitant arithmetic task. Cortex. **168** , 114–129, (10.1016/j.cortex.2023.08.005) 37708762

[5] Mulders D , de Bodt C , Lejeune N , Courtin A , Liberati G , Verleysen M , Mouraux A . 2020 Dynamics of the perception and EEG signals triggered by tonic warm and cool stimulation. PLoS ONE **15** , e0231698. (10.1371/journal.pone.0231698) 32324752 PMC7179871

[6] Liberati G , Algoet M , Santos SF , Ribeiro-Vaz JG , Raftopoulos C , Mouraux A . 2019 Tonic thermonociceptive stimulation selectively modulates ongoing neural oscillations in the human posterior insula: evidence from intracerebral EEG. Neuroimage **188** , 70–83, (10.1016/j.neuroimage.2018.11.059) 30529399

[7] Atlas LY , Wager TD . 2012 How expectations shape pain. Neurosci. Lett. **520** , 140–148. (10.1016/j.neulet.2012.03.039) 22465136

[8] Atlas LY , Bolger N , Lindquist MA , Wager TD . 2010 Brain mediators of predictive cue effects on perceived pain. J. Neurosci. **30** , 12964–12977. (10.1523/JNEUROSCI.0057-10.2010) 20881115 PMC2966558

[9] Hauck M , Lorenz J , Zimmermann R , Debener S , Scharein E , Engel AK . 2007 Duration of the cue-to-pain delay increases pain intensity: a combined EEG and MEG study. Exp. Brain Res. **180** , 205–215. (10.1007/s00221-007-0863-x) 17287993

[10] Jepma M , Koban L , van Doorn J , Jones M , Wager TD . 2018 Behavioural and neural evidence for self-reinforcing expectancy effects on pain. Nat. Hum. Behav. **2** , 838–855. (10.1038/s41562-018-0455-8) 31558818 PMC6768437

[11] Keltner JR , Furst A , Fan C , Redfern R , Inglis B , Fields HL . 2006 Isolating the modulatory effect of expectation on pain transmission: a functional magnetic resonance imaging study. J. Neurosci. **26** , 4437–4443. (10.1523/JNEUROSCI.4463-05.2006) 16624963 PMC6674009

[12] Lobanov OV , Zeidan F , McHaffie JG , Kraft RA , Coghill RC . 2014 From cue to meaning: brain mechanisms supporting the construction of expectations of pain. Pain **155** , 129–136. (10.1016/j.pain.2013.09.014) 24055334 PMC3947355

[13] Albu S , Meagher MW . 2016 Expectation of nocebo hyperalgesia affects EEG alpha-activity. Int. J. Psychophysiol. **109** , 147–152, (10.1016/j.ijpsycho.2016.08.009) 27562424

[14] Huneke NTM , Brown CA , Burford E , Watson A , Trujillo-Barreto NJ , El-Deredy W , Jones AKP . 2013 Experimental placebo analgesia changes resting-state alpha oscillations. PLoS ONE **8** , e78278. (10.1371/journal.pone.0078278) 24147129 PMC3795660

[15] Babiloni C , Brancucci A , Del Percio C , Capotosto P , Arendt-Nielsen L , Chen ACN , Rossini PM . 2006 Anticipatory electroencephalography alpha rhythm predicts subjective perception of pain intensity. J. Pain **7** , 709–717. (10.1016/j.jpain.2006.03.005) 17018331

[16] Franciotti R , Ciancetta L , Della Penna S , Belardinelli P , Pizzella V , Romani GL . 2009 Modulation of alpha oscillations in insular cortex reflects the threat of painful stimuli. Neuroimage **46** , 1082–1090. (10.1016/j.neuroimage.2009.03.034) 19327401

[17] Strube A , Rose M , Fazeli S , Büchel C . 2021 The temporal and spectral characteristics of expectations and prediction errors in pain and thermoception. Elife **10** , e62809. (10.7554/eLife.62809) 33594976 PMC7924946

[18] Nickel MM , Tiemann L , Hohn VD , May ES , Gil Ávila C , Eippert F , Ploner M . 2022 Temporal-spectral signaling of sensory information and expectations in the cerebral processing of pain. Proc. Natl Acad. Sci. USA **119** , e2116616119. (10.1073/pnas.2116616119) 34983852 PMC8740684

[19] Bott FS , Nickel MM , Hohn VD , May ES , Gil Ávila C , Tiemann L , Gross J , Ploner M . 2023 Local brain oscillations and interregional connectivity differentially serve sensory and expectation effects on pain. Sci. Adv. **9** , eadd7572. (10.1126/sciadv.add7572) 37075123 PMC10115421

[20] Koyama T , McHaffie JG , Laurienti PJ , Coghill RC . 2005 The subjective experience of pain: where expectations become reality. Proc. Natl Acad. Sci. USA **102** , 12 950–12 955. (10.1073/pnas.0408576102) PMC1200254 16150703

[21] R Core Team . 2021 R: a language and environment for statistical computing. Vienna, Austria: R Foundation for Statistical Computing. See https://www.R-project.org/.

[22] Frigo M , Johnson SG . FFTW: an adaptive software architecture for the FFT. In 1998 IEEE Int. Conf. on Acoustics, Speech, and Signal Processing, Seattle, WA, USA, pp. 493–498. 10.1111/2041-210X.12504.

[23] Dienes Z . 2021 Obtaining evidence for no effect. Collabra. Psychol. **7** . (10.1525/collabra.28202)

[24] Regan D . 1989 Human brain electrophysiology. In Evoked potentials and evoked magnetic fields in science and medicine. (10.1136/bjo.74.4.255-a)

[25] Mouraux A , Iannetti GD , Colon E , Nozaradan S , Legrain V , Plaghki L . 2011 Nociceptive steady-state evoked potentials elicited by rapid periodic thermal stimulation of cutaneous nociceptors. J. Neurosci. **31** , 6079–6087. (10.1523/JNEUROSCI.3977-10.2011) 21508233 PMC6632977

[26] Colon E , Legrain V , Mouraux A . 2014 EEG frequency tagging to dissociate the cortical responses to nociceptive and nonnociceptive stimuli. J. Cogn. Neurosci. **26** , 2262–2274. (10.1162/jocn_a_00648) 24738772 PMC5321242

[27] Hyvärinen A , Oja E . 2000 Independent component analysis: algorithms and applications. Neural Netw. **13** , 411–430. (10.1016/s0893-6080(00)00026-5) 10946390

[28] Bell AJ , Sejnowski TJ . 1995 An information-maximization approach to blind separation and blind deconvolution. Neural Comput. **7** , 1129–1159. (10.1162/neco.1995.7.6.1129) 7584893

[29] Bland JM , Altman DG . 1996 Statistics notes: transforming data. BMJ **312** , 770–770. (10.1136/bmj.312.7033.770) 8605469 PMC2350481

[30] Cook RD . 1977 Detection of influential observation in linear regression. Technometrics **19** , 15–18. (10.1080/00401706.1977.10489493)

[31] Allen M , Poggiali D , Whitaker K , Marshall TR , van Langen J , Kievit RA . 2019 Raincloud plots: a multi-platform tool for robust data visualization. Wellcome Open Res. **4** , 63. (10.12688/wellcomeopenres.15191.2) 31069261 PMC6480976

[32] Schmidt R , Schmelz M , Ringkamp M , Handwerker HO , Torebjörk HE . 1997 Innervation territories of mechanically activated C nociceptor units in human skin. J. Neurophysiol. **78** , 2641–2648. (10.1152/jn.1997.78.5.2641) 9356413

[33] Treede RD , Meyer RA , Campbell JN . 1990 Comparison of heat and mechanical receptive fields of cutaneous C-fiber nociceptors in monkey. J. Neurophysiol. **64** , 1502–1513. (10.1152/jn.1990.64.5.1502) 2283538

[34] Tillman DB , Treede RD , Meyer RA , Campbell JN . 1995 Response of C fibre nociceptors in the anaesthetized monkey to heat stimuli: correlation with pain threshold in humans. J. Physiol. **485** , 767–774. (10.1113/jphysiol.1995.sp020767) 7562615 PMC1158042

[35] Strube A , Horing B , Rose M , Büchel C . 2023 Agency affects pain inference through prior shift as opposed to likelihood precision modulation in a Bayesian pain model. Neuron **111** , 1136–1151.(10.1016/j.neuron.2023.01.002) 36731468 PMC10109109

[36] Thomaidou MA , Blythe JS , Houtman SJ , Veldhuijzen DS , van Laarhoven AIM , Evers AWM . 2021 Temporal structure of brain oscillations predicts learned nocebo responses to pain. Sci. Rep. **11** , 9807. (10.1038/s41598-021-89368-0) 33963251 PMC8105329

[37] Tiemann L , May ES , Postorino M , Schulz E , Nickel MM , Bingel U , Ploner M . 2015 Differential neurophysiological correlates of bottom-up and top-down modulations of pain. Pain **156** , 289–296. (10.1097/01.j.pain.0000460309.94442.44) 25599450

[38] Wager TD , Matre D , Casey KL . 2006 Placebo effects in laser-evoked pain potentials. Brain Behav. Immun. **20** , 219–230. (10.1016/j.bbi.2006.01.007) 16571371 PMC3735137

[39] Bradley C , Bastuji H , Garcia-Larrea L . 2017 Evidence-based source modeling of nociceptive cortical responses: a direct comparison of scalp and intracranial activity in humans. Hum. Brain. Mapp. **38** , 6083–6095. (10.1002/hbm.23812) 28925006 PMC6866722

[40] Leu C , Glineur E , Liberati G . 2024 Data from: Cue-based modulation of pain stimulus expectation: do ongoing oscillations reflect changes in pain perception? A registered report. Figshare. (10.6084/m9.figshare.c.7249296) PMC11296059 39100172

